# Draft Genome Sequence of the Psychrotolerant Bacterium Kurthia sibirica ATCC 49154^T^

**DOI:** 10.1128/MRA.00841-18

**Published:** 2018-08-09

**Authors:** Abigail E. Goen, Tyler Silverwood, Aria Underriner, Ariel M. Trachtenberg, Carolyn Kelley, Kyle S. MacLea

**Affiliations:** a Biology Program, University of New Hampshire, Manchester, New Hampshire, USA; b Biotechnology Program, University of New Hampshire, Manchester, New Hampshire, USA; c Biomedical Science and Technology Program, Seacoast School of Technology, Exeter, New Hampshire, USA; d Department of Life Sciences, University of New Hampshire, Manchester, New Hampshire, USA; University of Tennessee at Knoxville

## Abstract

The aerobic, Gram-positive, psychrotolerant bacterium Kurthia sibirica was first isolated from the stomach and intestinal contents of the Magadan mammoth recovered from the permafrost in eastern Siberia in 1977. K. sibirica was sequenced, and the predicted genome size is 3,496,665 bp, with 36.42% G+C content.

## ANNOUNCEMENT

Kurthia sibirica strain 13-2^T^ (ATCC 49154, CCM 3477, VKM B-1549) is an aerobic Gram-positive bacterium, motile via flagella, from the *Planococcaceae* family. This strain was initially isolated from the intestinal contents and stomach of the Magadan mammoth that had recently been removed from the permafrost in eastern Siberia in 1977 by Belikova and colleagues (1, 2). Other species in the Kurthia genus have been isolated from a variety of meats and meat products and spoiled food, as well as from the feces of certain domestic animals (3–5), and have been identified as pathogens (6).

Members of the Kurthia genus are Gram-positive aerobes that do not ferment glucose. They are usually motile with peritrichous flagella and form regular, unbranched rods with rounded ends. In older cultures, these bacteria form coccoid-like cells due to fragmentation of rods (1). K. sibirica differs from other Kurthia species in that it has high-vitamin nutritional requirements, cannot grow in a 7% NaCl environment, exhibits phosphatase activity, synthesizes a yellow pigment, has low DNA-DNA hybridization (<45%) compared with that of Kurthia zopfii and Kurthia gibsonii, and lacks coccoid formation in older cultures (1). K. sibirica can also grow in pH ranges of 5.5 to 9.5 and is able to produce acid from fructose and glycerol but not from ethanol or ribose (1).

K. sibirica ATCC 49154^T^ in lyophilized form was purchased from the ATCC (Manassas, VA, USA). It was rehydrated in tryptic soy broth and incubated in a shaking incubator at 25°C for 48 hours. Two drops of inoculated broth were spread onto tryptic soy agar and incubated at 26°C. A single colony was selected and plated onto brain heart infusion (BHI) agar and struck for isolates, one colony of which was then used to inoculate BHI broth and to obtain genomic DNA (gDNA) using the Qiagen DNA minikit (Valencia, CA, USA). A double-stranded DNA (dsDNA) broad-range assay kit (DeNovix, Wilmington, DE, USA) was used to check the integrity of the DNA. Purified gDNA was fragmented and tagged with sequence adapters using a Nextera DNA library prep kit (Illumina, San Diego, CA, USA) and subsequently sequenced on an Illumina HiSeq 2500 instrument (Hubbard Center for Genome Studies, Durham, NH, USA). With the HiSeq 2500 instrument, we generated 250-bp paired-end read sequences, which were bioinformatically trimmed prior to assembly and gene analysis using Trimmomatic to remove aberrant or mistagged fragments (7). These trimmed reads were then assembled using SPAdes version 3.11.1 with default settings (8). A total of 3,843,530 reads making up 960,882,500 bp of sequence were assembled into 79 total contigs after removal of small contigs and contaminants. This represents 274× average coverage of a total sequence length of 3,496,665 bp. The largest contig was 361,432 bp, with an *N*_50_ value of 138,251 bp and a G+C content of 36.42%. The assembled contigs were annotated using the NCBI Prokaryotic Genome Annotation Pipeline (PGAP) process (9) and found to have a total of 3,400 genes, 3,250 protein-coding sequences, 69 pseudogenes, 7 rRNAs, and 69 tRNAs.

### Data availability.

The Kurthia sibirica ATCC 49154^T^ whole-genome shotgun sequence (WGS) project has been deposited in DDBJ/ENA/GenBank under accession number QFVR00000000. The version described in this paper is the first version, QFVR01000000.
